# Empirical antibiotic prescribing in COVID-19 patients: patterns and predictors in a resource-constrained Central Indian setting

**DOI:** 10.3389/frabi.2026.1826199

**Published:** 2026-05-20

**Authors:** Manju Purohit, Deepali Pathak, Devendra Baghel, Jitendra Jat, Ashish Sharma, Ashish Pathak

**Affiliations:** 1 Department of Pathology, Ruxmaniben Deepchand Gardi Medical College, Ujjain, Madhya Pradesh, India; 2 Department of Global Public Health, Health Systems and Policy (HSP): Medicines, Focusing Antibiotics, Karolinska Institutet, Stockholm, Sweden; 3 Department of Pediatrics, Ruxmaniben Deepchand Gardi Medical College, Ujjain, Madhya Pradesh, India; 4 Department of Medicine, R. D. Gardi Medical College, Ujjain, India

**Keywords:** antimicrobial resistance, antimicrobial stewardship, COVID-19, C-reactive protein, DU90%, empirical antibiotic prescription, India, resource-limited setting

## Abstract

**Background:**

Empirical antibiotic prescribing during the COVID-19 pandemic may have accelerated antimicrobial resistance, particularly in resource-constrained settings with limited diagnostic and stewardship capacity. This study characterized antibiotic prescribing patterns and identified predictors among hospitalized COVID-19 patients in rural Central India.

**Methods:**

This retrospective observational study included 951 laboratory-confirmed COVID-19 patients (aged ≥18 years, hospitalized ≥48 hours) admitted between March 2020 and December 2021. Antibiotic use was assessed using WHO ATC/DDD methodology and Drug Utilization 90% (DU90%) analysis. Predictors of antibiotic prescription were evaluated using multivariable logistic regression. Empirical prescribing was defined as antibiotic initiation without microbiological confirmation.

**Results:**

Among 951 patients (mean age 53 ± 15.8 years; 64% male), 54% had ≥1 comorbidity and 74% required oxygen; 20% required mechanical ventilation. Antibiotics were prescribed to 90% (858/951); 46% received two agents and 8% ≥3 agents. The DU90% segment consisted of WHO “Watch” antibiotics, mainly amoxicillin-clavulanate (40.87 g/100 patient-days), doxycycline (18.26), and azithromycin (17.12). However, the use of meropenem (4.04 DDD gm/100 patients) a WHO Reserve group carbapenem is particularly alarming. Elevated C-reactive protein (>10 mg/dL) was the strongest predictor of antibiotic use (adjusted OR 29.28, 95% CI 17.53–48.89; p<0.001). Antibiotic recipients had longer median hospital stays (11 vs 8 days). Overall mortality was 26%.

**Conclusions:**

Empirical antibiotic use was extremely high and driven by WHO “Watch” and “reserve” group agents, largely associated with elevated CRP in the absence of culture testing. Strengthening diagnostics and stewardship programs is urgently needed to reduce unnecessary broad-spectrum antibiotic exposure.

## Introduction

The global surge of coronavirus disease 2019 presented unprecedented challenges to healthcare systems worldwide, particularly in resource-limited regions, necessitating rapid adaptation of existing treatment protocols (Mondal et al., 2022). This emergent situation led to the adoption of empirical treatment strategies, including widespread antibiotic prescribing, despite the viral etiology of the disease (Stoichitoiu et al., 2022). This practice gained prominence because the clinical differentiation between primary viral pneumonia and potential secondary bacterial co-infections or superinfections remained challenging, especially in settings with limited diagnostic capabilities (Stevens et al., 2021).

The pervasive empirical antibiotic administration during the COVID-19 pandemic has been a key driver of antimicrobial resistance, which has been most common in regions with nascent or inadequately implemented antibiotic stewardship programs (Mohammed et al., 2020). The high rates of antibiotic prescribing during the COVID-19 pandemic, often without microbiological confirmation of bacterial co-infection, underscore the urgent need for robust antibiotic stewardship (Martin et al., 2023). The increase in broad-spectrum antibiotic prescriptions for COVID-19 management and the high prevalence of multidrug-resistant bacterial pathogens, such as Klebsiella pneumoniae and Acinetobacter baumannii, reported in similar settings, reinforce the need for such programs (Kar et al., 2023).

While the broad impact of empirical antibiotic prescription in COVID-19 is recognized, detailed, context-specific data on prescribing patterns and associated factors are crucial, particularly for resource-constrained settings in Central India. This study aims to characterize the patterns of antibiotic prescription among COVID-19 patients admitted to a single center located in Central India and identify their associated factors, thereby contributing to a more nuanced understanding of antimicrobial prescribing practices in a resource-constrained environment.

## Methodology

### Study design and setting

This retrospective study was conducted among COVID-19 patients admitted to the inpatient wards of CR Gardi Hospital, attached to R.D. Gardi Medical College, Ujjain, between March 2020 and December 2021. CRGH served as a tertiary care referral center for COVID-19 cases in Ujjain district. R.D. Gardi Medical College is located in a rural part of Ujjain district in Central India and was a designated hospital for the treatment of patients with COVID-19 during the study period. Madhya Pradesh is India’s second-largest state by area, with 72 million inhabitants and approximately 72% of the population residing in rural areas (Department of Farmer Welfare and Agriculture Development MP, 2026). The study is reported in accordance with the Strengthening the Reporting of Observational Studies in Epidemiology (STROBE) guidelines.

### Sampling strategy

This study included all consecutive patients admitted to CR Gardi Hospital after a diagnosis of COVID-19. A total of 951 cases were recorded during the study period. The included patients were confirmed COVID-19-positive based on a) polymerase chain reaction (PCR) or b) COVID-19 antigen test as per the National treatment Guidelines of India (Kumar et al., 2021). Patients were eligible for inclusion if they were aged 18 years or older, had confirmed SARS-CoV-2 infection, and were hospitalized for at least 48 hours. Patients were excluded if SARS-CoV-2 infection was not laboratory confirmed, if medical records were incomplete, if C-reactive protein (CRP) or total leukocyte count (TLC) values at admission were missing, or if discharge or outcome data were unavailable. Consecutive sampling was employed to minimize selection bias. For this study, empirical antibiotic prescribing was defined as initiation of systemic antibiotic therapy within the first 24 hours of admission, before any microbiological culture results were available or sent.

### Data collection

Clinical variables included presenting symptoms, comorbidities, requirement for supplemental oxygen, need for mechanical ventilation, and documented complications such as acute respiratory distress syndrome (ARDS), septic shock, acute kidney injury, and other in-hospital events. Comorbid conditions recorded were hypertension, diabetes mellitus, cardiovascular disease, tuberculosis, chronic kidney disease, chronic liver disease, and other documented chronic illnesses. The specific COVID-19 wave was also extracted from medical records (**Supplementary File 1**: data collection form used in the study). Data extraction was performed by trained personnel using a standardized data collection form to ensure consistency and accuracy. Patients were categorized into two age groups: 20–40 years and ≥40 years. Patients were also classified for duration of hospital stay as ≥15 days and <15 days. Patient outcome was defined as either death or discharge from the hospital.

The primary outcome of interest was antibiotic prescription during hospital stay, defined as receipt of at least one systemic antibiotic at any time during hospitalization. Secondary measures included the number of antibiotics prescribed, duration of hospital stay, and in-hospital mortality. The following laboratory investigation reports were extracted from patient files and used to correlate with antibiotic prescribing: a) complete blood count (CBC), measured using a five-part automated coulter counter (XS-800i, Sysmex India Pvt. Ltd., India); a leukocyte count of >11,000 cells/L was considered indicative of a suspected secondary bacterial infection. b) quantitative CRP measurement, using Vitros CRP slides on the Vitros 3600 Chemistry Analyzer (Ortho Clinical Diagnostics, Johnson & Johnson, USA); a value >10 mg/dL was considered indicative of a suspected secondary bacterial infection (Pink et al., 2021). These cut-off values for TLC and CRP are based on widely accepted clinical indicators for bacterial infection in acute setting (Pink et al., 2021). CBC and CRP levels for all patients were performed within 24 h of hospital admission. The severity of COVID-19 was categorized based on WHO clinical management guidelines, ranging from asymptomatic to critical, taking into account factors such as oxygen saturation, respiratory rate, and chest X-ray findings (Ul Mustafa et al., 2024). Microbiological cultures (e.g., blood, sputum, or urine) were not sent due to the limited diagnostic infrastructure available in this resource-constrained setting. Consequently, clinical decisions regarding antibiotic initiation were predominantly based on clinical suspicion and inflammatory markers rather than confirmed bacterial etiology. Missing data for variables other than CRP and TLC were handled using complete-case analysis, with records excluded only from the specific analyses where values were missing.

### Antibiotic prescription pattern

Empirical antibiotic prescribing was defined as the initiation of systemic antibiotics within 24 hours of the hospital admission, without microbiological confirmation.

Antibiotic prescription data were analyzed and categorized based on the pharmacological class of antibiotics, type, dosage form, route of administration, and the anatomical therapeutic chemical (ATC) classification codes, as defined by the World Health Organization (WHO) (Mondal et al., 2022). The classification system was referenced from the WHO Collaborating Centre for Drug Statistics Methodology (Mondal et al., 2022). Antibiotic prescription was categorized according to the ATC groups and codes. Total antibiotic consumption was calculated over a specified period by dividing the total drug administered (in gram) by the defined daily dose (DDD), length of stay (LOS), and study duration. The standardized metric for evaluation was defined as DDD per 100 patient-days, calculated using the following formula:


$$\text{DDD}=\frac{\text{Number of grams of antibiotics used by COVID}-19\text{ patient}}{\text{WHO DDD Standard }(\text{gram})}\times \frac{100}{\text{Total LOS}}$$

Drug Utilization 90% (DU90%) for the antibiotics prescribed was also calculated (Bergman et al.,1998), which helped assess whether the commonly used medicines (by volume) are consistent with those recommended in standard treatment guidelines (Bergman et al.,1998). This analysis allows for a comparison between actual prescribing practices and evidence-based recommendations, highlighting potential areas for intervention in antibiotic stewardship (Stevens et al., 2021). DU90% calculation also helps in identifying deviations from optimal prescribing, thereby providing insights to refine targeted educational interventions or implement policy adjustments to mitigate the escalating threat of antimicrobial resistance (Stevens et al., 2021).

### Bias minimization

Selection bias was minimized through consecutive inclusion of all eligible patients during the study period. Standardized laboratory thresholds were applied uniformly to reduce misclassification bias. As the study was retrospective and based on routinely collected hospital data, the possibility of residual confounding cannot be excluded.

### Data analysis

The data were entered into EpiData Entry (version 3.1) and then transferred to Stata 16.0 (Stata Corp. College Station, Texas, USA) for statistical analysis. Continuous variables were summarized as mean with standard deviation or median with interquartile range, depending on distribution. Categorical variables were presented as frequencies and percentages. The prevalence of antibiotic prescription was estimated with corresponding 95% confidence intervals.

The primary outcome variable was empiric antibiotic prescription (within 24 hr of admission) and prescription anytime during hospitalization, categorized as a binary variable. Univariable logistic regression analysis was conducted to examine associations between each independent variable and antibiotic prescription. Crude odds ratios with 95% confidence intervals were calculated. The independent variables included various signs and symptoms associated with COVID-19 and laboratory parameters, including CRP and CBC. In addition, parameters of healthcare system utilization, such as oxygen support, mechanical ventilation, and duration of hospital stay, were recorded. Crude ORs were calculated using two-by-two tables. Chi-square tests were used to determine the statistical significance (α = 0.05). Two-sided p-values less than 0.05 were considered statistically significant. Patients with missing CRP or TLC values were excluded from regression analysis. As missing data for included variables were minimal, complete-case analysis was performed.

Stepwise multivariate logistic regression with backward elimination was used to develop the model. In bivariate analyses, a p-value of <0.1 was considered significant for entry into the model, with antibiotic prescription as the outcome variable. The analysis was started with the full model, then the variable with highest p-value was removed, and the model was revised and refitted with remaining predictors. The procedure was repeated until the p value of all the predictor variables was less than 0.1 except for age and gender; this was designated as the final model. Adjusted odds ratios and their respective 95% confidence intervals (CI) were then calculated from the final model. A p-value of < 0.05 was considered significant in the final model. Model discrimination was assessed with an area under the receiver operating characteristic curve (AUC). Calibration was assessed using the Hosmer–Lemeshow goodness-of-fit test.

### Ethical considerations

The Institutional Ethics Committee approved the study (Approval No. IEC Ref No 22/2020). The procedures followed were in accordance with the ethical standards set by the institutional ethics committee and with the Declaration of Helsinki.

## Results

**Figure 1:**
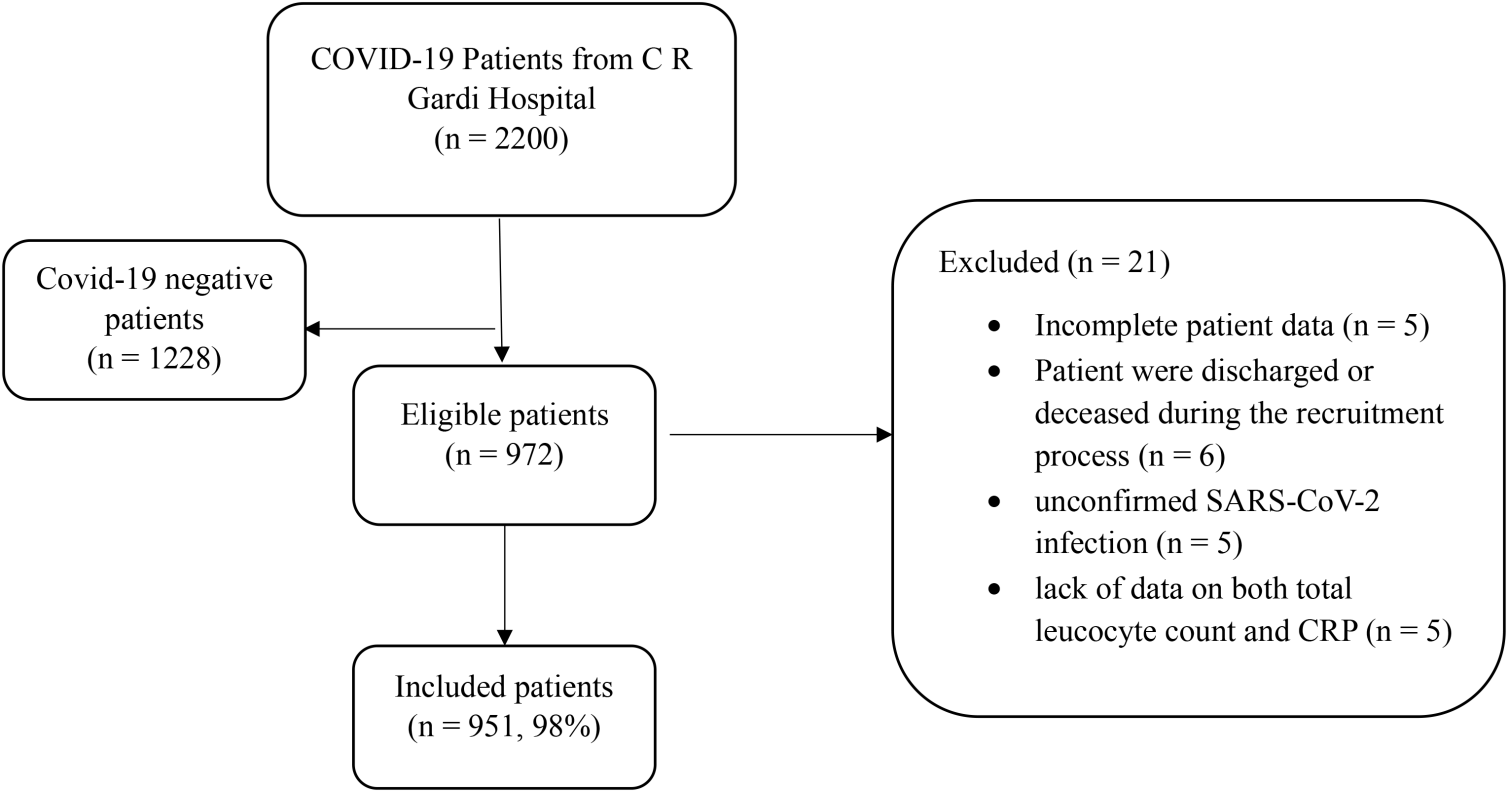
The recruitment process of participants included in the study.

A total of 951 COVID-19 patients were included in the study. Recruitment process for the participants is shown in **Figure 1**.

**Table 1** details the demographic and clinical characteristics of the 951 hospitalized COVID-19 patients across the first to third waves. The number of patients in the first, second, and third waves were 331 (35%), 514 (54%), 106 (11%), respectively. The mean age (± SD) was 53 ± 15.83 years, with 610 (64%) patients being male and 341 (36%) females. The median time from symptom onset to admission was 3 days. A total of 512 (54%) patients had comorbidities, with hypertension (42%) and diabetes mellitus (23%) being the most common. Other comorbidities are presented in **Table 1**. A total of 934 (98%) patients were symptomatic at admission, while only 17 (2%) were asymptomatic.

**Table 1:** Demographic and clinical characteristics of hospitalized COVID-19 patients across the first to third waves.

Characteristic	Total (n = 951)
Age (yr.), mean ± SD	53 ± 15.83
Age categories (yr.)	
20-40	219 (23)
>40	732 (77)
Gender	
Male	610 (64)
Female	341 (36)
Days from symptom onset to admission, median (IQR)	3 (2 - 4)
One or more comorbidities	
Present	512 (54)
Absent	439 (46)
Specific comorbidities	
Hypertension	397 (42)
Diabetes mellitus	216 (23)
Chronic cardiac disease	26 (3)
Hypothyroidism	14 (1)
Asthma	12 (1)
Chronic pulmonary disease	4 (1)
Tuberculosis	2 (1)
Chronic kidney disease	3 (1)
Chronic liver disease	2 (1)
Malignancy	3 (1)
Stroke	3 (1)
Obesity	9 (1)
HIV infection	2 (1)
Symptomatic status	
Symptomatic	934 (98)
Asymptomatic	17 (2)

Data are presented as number (%) unless otherwise indicated. SD, standard deviation; IQR, interquartile range.

**Table 2** shows the frequency of symptoms and their association with antibiotic prescription among 951 patients included in the study. A total of 858 patients (90%; 95% CI 88% – 92%) received one antibiotic. The number of antibiotics prescribed per patient was as follows: one antibiotic to 343 patients (36%), two antibiotics to 437 patients (46%), three antibiotics to 60 patients (6%), and four or more antibiotics to 18 patients (2%).

**Table 2:** Frequency of sign and symptoms and their association with antibiotic prescription among hospitalized COVID−19 patients (n = 951).

Variables	Total (n = 951)	Prescribed antibiotic		OR	95% CI	p-value
		No (n=93, %)	Yes (n=858, %)			
History of fever						
No	98 (10)	19 (19)	79 (81)	R	R	–
Yes	853 (90)	74 (9)	779 (91)	2.53	1.45 – 4.40	0.001
Respiratory sign/symptom						
Dry cough						
No	344 (36)	46 (13)	298 (87)	R	R	–
Yes	607 (64)	47 (8)	560 (92)	1.83	1.19 – 2.82	0.005
Shortness of breath						
No	438 (46)	52 (12)	386 (88)	R	R	–
Yes	513 (54)	41 (8)	472 (92)	1.55	1.00 – 2.38	0.046
Sore throat						
No	561 (59)	66 (12)	495 (88)	R	R	–
Yes	390 (41)	27 (7)	363 (93)	1.79	1.12 – 2.86	0.014
Rhinorrhea						
No	811 (85)	81 (10)	730 (90)	R	R	–
Yes	140 (15)	12 (9)	128 (91)	1.18	0.62 – 2.23	0.603
Cough with sputum						
No	874 (92)	89 (10)	785 (90)	R	R	–
Yes	77 (8)	4 (5)	73 (95)	2.06	0.73 – 5.79	0.166
General sign/symptom						
Headache						
No	659 (69)	67 (10)	592 (90)	R	R	–
Yes	292 (31)	26 (9)	266 (91)	1.15	0.71 – 1.86	0.546
Fatigue						
No	713 (75)	75 (11)	638 (89)	R	R	–
Yes	238 (25)	18 (8)	220 (92)	1.43	0.83 – 2.45	0.186
Arthralgia						
No	808 (85)	81 (10)	727 (90)	R	R	–
Yes	143 (15)	12 (8)	131 (92)	1.21	0.64 – 2.29	0.545
Limitation of activities						
No	93 (10)	47 (51)	46 (49)	R	R	–
Yes	858 (90)	372 (43)	486 (57)	1.33	0.86 – 2.04	0.186
Cardiovascular system						
Chest pain						
No	929 (98)	91 (10)	838 (90)	R	R	–
Yes	22 (2)	2 (9)	20 (91)	1.08	0.24 – 4.72	0.912
Cold hands/feet						
No	944 (99)	91 (10)	853 (90)	R	R	–
Yes	7 (1)	2 (29)	5 (71)	0.26	0.05 – 1.39	0.117
Gastrointestinal symptoms						
Vomiting						
No	923 (97)	91 (10)	832 (90)	R	R	–
Yes	28 (3)	2 (7)	26 (93)	1.42	0.33 – 6.08	0.635
Abdominal pain						
No	932 (98)	91 (10)	841 (90)	R	R	–
Yes	19 (2)	2 (11)	17 (89)	0.91	0.20 – 4.04	0.912
Diarrhea						
No	941 (99)	91 (10)	850 (90)	R	R	–
Yes	10 (1)	2 (20)	8 (80)	0.42	0.08 – 2.04	0.288
Central nervous system						
Loss taste/altered taste						
No	760 (80)	81 (11)	679 (89)	R	R	–
Yes	191 (20)	12 (6)	179 (94)	1.77	0.94 – 3.33	0.072
Anosmia						
No	861 (91)	85 (10)	776 (90)	R	R	–
Yes	90 (9)	8 (9)	82 (91)	1.12	0.52 – 2.40	0.765
Weakness of limbs/inability to walk						
No	901 (95)	87 (10)	814 (90)	R	R	–
Yes	50 (5)	6 (12)	44 (88)	0.78	0.32 – 1.89	0.588
Seizures						
No	941 (99)	92 (10)	849 (90)	R	R	–
Yes	10 (1)	1 (10)	9 (90)	0.97	0.12 – 7.78	0.981

R, Reference category; OR, Odds Ratio; CI, Confidence Interval. P <0.05 was considered statistically significant % row percentage.

A total of 787 out of 951 patients (83%) were prescribed antibiotics within 24 hours of admission, representing empirical antibiotic prescriptions. A total of 71 patients (7%) were prescribed antibiotics at some point during their hospital stay.

**Figure 2:**
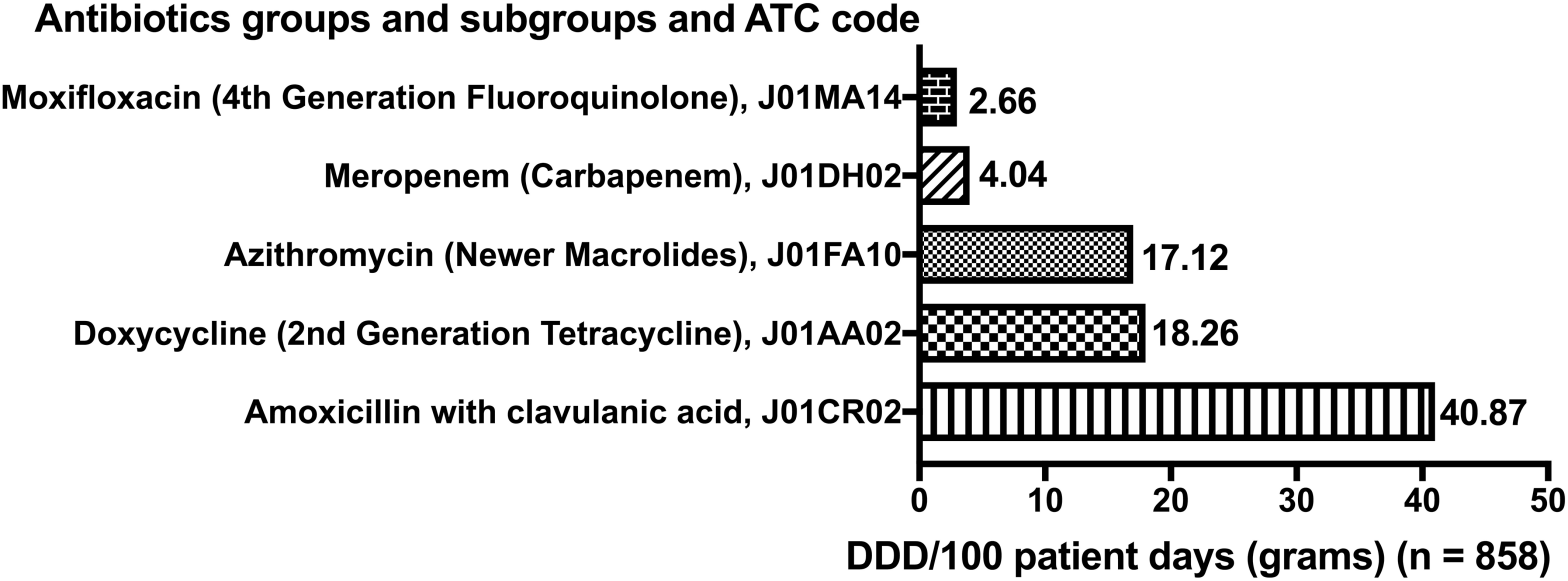
DU 90% analysis of antibiotic utilization by groups and subgroups (DDD per 100 patients, n = 858).

**Figure 2** shows the DU90% analysis of antibiotic prescription by groups and subgroups (DDD per 100 patients, n = 858). Amoxicillin with clavulanic acid, J01CR02 (40.87 g/100 patients), was the most common antibiotic prescribed, followed by doxycycline, J01AA02 (18.26 g/100 patients), least common was moxifloxacin, J01MA14 (2.66 g/100 patients).

**Table 3** shows the complications and outcomes among 951 patients hospitalized with COVID-19 across the first through third waves. Acute respiratory distress syndrome (ARDS) was the most common complication, observed in 531 patients (56%), followed by septic shock in 198 (21%). A total of 249 patients (26%) died due to COVID-19 related complications.

**Table 3:** Complications and outcome seen among hospitalized COVID-19 patients.

Complication/Outcome	Total (n = 951) n (%)	Mean (± SD)	Range
Complications			
Acute respiratory distress syndrome (ARDS)	531 (56)		
Septic shock	198 (21)		
Hyperglycemia	16 (2)		
Acute kidney injury	10 (1)		
Anemia	9 (1)		
Hospital acquired infection	6 (1)		
Liver dysfunction	2 (1)		
Lab Parameters			
CRP (C- reactive Protein)			
< 10 mg/dL	107 (11)	40.45 (± 41.42)	0.03 – 314.5
> 10 mg/dL	844 (89)		
TLC (Total Leukocyte Count)			
Leukopenia (<11000)	743 (78)	8409 (± 5294.54)	1254 – 46400
Leukocytosis (>11000)	208 (22)		
Outcomes			
Death	249 (26)		
Discharge	702 (74)		
Death by age category (n = 249)			
20–40 years (n = 219)	39 (16)		
>40 years (n = 732)	210 (84)		

Data are presented as number (%). ARDS, acute respiratory distress syndrome. Death by age category is shown as number of deaths (% of total deaths, n = 249).

Among the 858 patients who received antibiotics, 531 (56%) were diagnosed with ARDS. The most common antibiotics prescribed for ARDS included azithromycin (231.33 g/100 patients) and doxycycline (119.46 g/100 patients). A total of 198 (21%) patients were diagnosed with septic shock. The most commonly prescribed antibiotics in this subgroup were doxycycline (7.33 g/100 patients) and amoxicillin with clavulanic acid (7.33 g/100 patients).

**Table 4** presents parameters of healthcare system utilization during the pandemic among 951 hospitalized COVID-19 patients.

**Table 4:** Parameters of healthcare system utilization in pandemic among hospitalized COVID-19 patients.

Characteristics	Total (n = 951)	Mean (± SD)	Range
Requiring supplemental oxygen	706 (74)		
Requiring mechanical ventilation among those requiring oxygen supplementation (n = 706)	143 (20)		
Duration of hospital stay in days, median (IQR)	10 (6 - 15)		
Duration of hospital stay in days in discharged patients, median (IQR) (n = 702)	11 (8 - 15)		
Duration of hospital stay in days in deceased patients, median (IQR) (n = 249)	6 (3 - 11)		
Duration of hospital stay in days with antibiotic prescription	858 (90)	11.87 (± 7.86)	1 – 59
Duration of hospital stay in days with no antibiotic prescription	93 (10)	7.91 (± 5.69)	1 – 27

Data are presented as number (%) or median (interquartile range), as appropriate. IQR, interquartile range.

**Supplementary Table 1** shows the WHO classification of severity of Covid-19 disease in the patients included in the study, with 32% (n = 303) of the total patients having severe Covid-19 disease and a total of 25% (n = 249) deaths, indicating more than 50% of the patients had severe disease.

An association between C-reactive protein (CRP) levels and antibiotic prescription was also explored. Patients were stratified into two groups based on CRP levels: low (<10 mg/dL) (n=107, 11%) and high (>10 mg/dL) (n=844, 89%). A significantly higher proportion of patients with high CRP levels received antibiotics (96%) compared to those with low levels (45%) (OR 29.28, 95% CI 17.53 – 48.89; p < 0.001).

There was no statically significant relationship between antibiotic prescription in patients with leukopenia or leukocytosis compared to those with normal TLC. (OR – 1.27, 95% CI 0.82 – 1.96; p = 0.279).

**Table 5** shows comorbid conditions such as hypertension, diabetes, chronic cardiac disease, hypothyroidism, and asthma were not significantly associated with antibiotic use.

**Table 5:** Association of comorbidities with antibiotic prescription among study participants (n = 951).

Variables	Total (n = 951)	Antibiotic prescribed		OR	95% CI	p-value
		No (n = 93) (n, %)	Yes (n = 858) (n, %)			
Comorbidities						
No	93 (10)	41 (44)	52 (56)	R	R	–
Yes	858 (90)	398 (46)	460 (54)	1.09	0.71 – 1.68	0.673
Hypertension						
No	93 (10)	58 (62)	35 (38)	R	R	–
Yes	858 (90)	496 (58)	362 (42)	1.20	0.77 – 1.87	0.398
Diabetes mellitus						
No	93 (10)	71 (76)	22 (24)	R	R	–
Yes	858 (90)	664 (77)	194 (23)	0.94	0.56 – 1.56	0.819
Chronic cardiac disease						
No	93 (10)	89 (96)	4 (4)	R	R	–
Yes	858 (90)	836 (97)	22 (3)	0.58	0.19- 1.73	0.335
Hypothyroidism						
No	93 (10)	91 (98)	2 (2)	R	R	–
Yes	858 (90)	846 (99)	12 (1)	0.64	0.14 – 2.92	0.570
Asthma						
No	93 (10)	90 (97)	3 (3)	R	R	–
Yes	858 (90)	849 (99)	9 (1)	0.31	0.08 – 1.19	0.090

### Logistic regression analysis: univariate analysis

The following independent variables were associated with antibiotic prescription in univariate analysis: Patients with a history of fever, the presence of dry cough, shortness of breath, sore throat and patients diagnosed with ARDS were significantly associated with antibiotic prescription.

### Multivariate logistic regression analysis

**Table 6** presents the results of multivariable logistic regression analysis of factors associated with antibiotic prescription. Age (continuous variable) was significantly associated with antibiotic prescription with each one-year increase associated with a 1% increase in the odds (AOR = 1.01; 95% CI: 1.00–1.03; p = 0.022). Patients presenting with cough with sputum and elevated CRP levels (>10 mg/dL) were associated with antibiotic prescription in the final model.

**Table 6:** Crude and adjusted logistic regression analysis of factors associated with antibiotic prescription (n = 951).

Variables	Total (n = 951)	Prescribed antibiotic		Crude OR (95% CI)	p-value	Adjusted OR (95% CI)	p-value
		No (n = 93)	Yes (n = 858)				
Mean(± SD)	Mean(± SD)	Mean(± SD)
**Age in years**	53.92(± 15.83)	52.05(± 17.11)	54.12(± 15.69)	1.00 (0.99 – 1.02)	0.681	1.01 (1.00 – 1.03)	0.022
	(n, %)	(n, %)	(n, %)				
Gender							
Male	610 (64)	60 (10)	550 (90)	R	–	R	
Female	341 (36)	33 (10)	308 (90)	1.01 (0.65 – 1.59)	0.937	1.20 (0.69 – 2.08)	0.505
Cough with sputum							
No	874 (92)	89 (10)	785 (90)	R	–	R	
Yes	77 (8)	4 (5)	73 (95)	2.06 (0.73 – 5.79)	0.166	3.84 (1.17 – 12.63)	0.026
CRP							
< 10 mg/dL	107 (11)	59 (63)	34 (37)	R	–	R	–
> 10 mg/dL	844 (89)	34 (4)	810 (96)	29.28 (17.53 – 48.89)	<0.001	34.59 (20.10 – 59.54)	<0.001

OR, Odds Ratio; CI, Confidence Interval; R, Reference category. Age was entered as a continuous variable (per one-year increase); CRP C-reactive Protein; p < 0.05 was considered statistically significant.

### Model performance

The final multivariable logistic regression model demonstrated good discrimination with an area under the receiver operating characteristic curve (AUC) of 0.81. Calibration assessed using the Hosmer–Lemeshow goodness-of-fit test showed no evidence of poor fit (p = 0.697). The model explained 30.8% of variation in antibiotic prescribing (Pseudo R² = 0.308). CRP >10 mg/dL was the dominant predictor of antibiotic prescription.

## Discussion

The findings from this single-center study in a resource-constrained setting from Central India offer crucial insights into the patterns of antibiotic prescription and its associated clinical factors in COVID-19 hospitalized patients. Our analysis indicates that broad-spectrum antibiotics, such as amoxicillin with clavulanic acid, doxycycline, and azithromycin, were predominantly prescribed which is consistent with the observations from other COVID-19 study cohorts that were administered empirical broad-spectrum antibiotics due to diagnostic uncertainties regarding bacterial co-infections (Sturza et al., 2023). Owing to challenges in distinguishing between severe COVID-19 disease and secondary bacterial infections, clinicians often administer broad-spectrum antibiotics, despite guidelines advocating against routine antibiotic prescription in moderate COVID-19 cases without clear bacterial infection (Bednarčuk et al., 2023; Hegazy et al., 2022). This empirical approach, though a potentially life-saving approach for critically ill patients, inadvertently contributes to the emergence of antibiotic-resistant strains (Manohar et al., 2020; Yahya, 2022; Ablakimova et al., 2023; Abdelsalam Elshenawy et al., 2024).

The lack of microbiological confirmation makes it difficult to differentiate between viral inflammation and true bacterial co-infection. This diagnostic uncertainty likely contributed to the very high rate of empirical antibiotic use observed in our study.

Frequent broad-spectrum antibiotic prescribing is particularly concerning given that bacterial co-infections in COVID-19 patients are rare, with studies reporting incidence rates ranging from 2% to 15% (Aldali et al., 2023; Sturza et al., 2023).

The practice of prescribing broad-spectrum antibiotics has increased the prevalence of antibiotic resistance, a global public health problem primarily prominent in low- and middle-income countries like India (Gandra et al., 2023). The observed antibiotic prescribing patterns in our study underscore the urgent need for robust antimicrobial stewardship programs in resource-constrained settings to optimize antibiotic prescribing practices, which is crucial to mitigate the emergence of antimicrobial resistance and preserve the efficacy of existing antimicrobial agents (Fésüs et al., 2024).

Our study used CRP and TLC as markers for bacterial infection in the absence of bacterial cultures. CRP levels greater than 10 mg/dL were significantly associated with antibiotic prescription, aligning with evidence that CRP is an acute phase reactant that rises non-specifically with inflammation, though often more dramatically in bacterial infections than in viral (Nemr et al., 2023). Conversely, the lack of a statistically significant association between TLC (leukopenia or leukocytosis) and antibiotic prescription in our cohort deviates from some established clinical norms, which often consider altered leukocyte counts as indicators of bacterial infection, thereby warranting further investigation into the utility of TLC as a sole biomarker for guiding antibiotic therapy in COVID-19 cases within such settings (Mairpady Shambat et al., 2022). CRP and TLC are poor surrogate markers for bacterial infection, especially in COVID-19 patients, where inflammatory responses can confound these markers and lead to inappropriate antibiotic prescription (Garg, 2021). This highlights a critical need for more specific and reliable biomarkers for bacterial co-infection in COVID-19 to guide judicious antibiotic prescribing (Ul Mustafa et al., 2024).

It is important to acknowledge that the strong association between elevated CRP and antibiotic prescription may reflect circularity in clinical decision-making. In this resource-limited setting, CRP was routinely used by clinicians as a surrogate marker to decide whether to start empirical antibiotics. Thus, CRP functioned more as a prescribing decision marker rather than an independent biological predictor of bacterial infection.

The clinical signs and symptoms that correlated with antibiotic prescribing in our study were fever which was associated with antibiotic prescribing. This aligns with previous research indicating that elevated temperature is often a primary trigger for antibiotic initiation by physicians, even when bacterial co-infection rates are low (Polat Yuluğ et al., 2024).

Among the respiratory signs and symptoms, dry cough, sore throat, and shortness of breath were associated with antibiotic prescribing. Similar findings have been reported in other studies, further emphasizing the challenge of differentiating viral pneumonia from bacterial co-infection based on clinical presentation alone (Ul Mustafa et al., 2024). This diagnostic ambiguity frequently leads to empirical antibiotic prescriptions for acute respiratory infections, despite the prevalence of viral etiologies that do not necessitate antibiotic intervention, thus exacerbating the challenge of antimicrobial resistance (Igirikwayo et al., 2024).

The median hospital stay of 10 days in our study aligns with findings from other Indian studies, although some international data report shorter durations, possibly reflecting differences in healthcare systems and patient profiles (Iwamoto et al., 2023). The length of hospital stay can be influenced by the severity of the disease and the availability of advanced therapeutic interventions like remdesivir or tocilizumab, which have been shown to expedite patient recovery and discharge times (Iwamoto et al., 2023). Patients receiving antiviral and antibiotic therapy tended to have longer hospital stays, indicating greater severity of the disease (Abduljabbar et al., 2022). This is further corroborated by evidence suggesting that bacterial and fungal co-infections are common complications in hospitalized patients with COVID-19, especially in settings with a high burden of infectious diseases (Vijay et al., 2021). Prolonged duration of hospitalization for patients with co-infections contributes to higher healthcare expenditures and places additional strain on resource-limited healthcare infrastructures (Galang-De Leon and Buensalido, 2022).

Although patients receiving antibiotics had longer hospital stays, this observation should not be interpreted as a causal effect of antibiotic therapy. It is more likely explained by confounding by indication — i.e., patients with greater disease severity were both more likely to receive antibiotics and had prolonged hospitalization.

In 2019, antimicrobial resistance, a global health crisis, was responsible for nearly 5 million deaths worldwide, and these data highlight the urgent need for multifaceted interventions, including improved surveillance, novel antimicrobial development, and stringent antimicrobial stewardship programs (Miteu et al., 2023; Al Jammal et al., 2024). Furthermore, alarming projections necessitate the implementation of comprehensive strategies that encompass not only judicious antibiotic prescription but also effective infection prevention and control measures (Micheli et al., 2023; Ahmed et al., 2024; Ho et al., 2025).

ATC DDD methodology used in the present study is a robust approach for quantifying antimicrobial consumption, enabling standardized comparisons across diverse settings and facilitating the identification of patterns that contribute to antimicrobial resistance (Nagassar et al., 2023). The inclusion of meropenem, a WHO Reserve group carbapenem, in the DU90% segment is particularly alarming as it indicates use of last-resort antibiotics in empirical therapy, further increasing the risk of antimicrobial resistance (Abdelsalam Elshenawy et al., 2023). Although this method can provide valuable insights into the quantity of antibiotics consumed, it does not fully capture the appropriateness of prescribing or the quality of antibiotic prescription, which are equally critical for effective antimicrobial stewardship.

Additionally, the DU90% methodology used in the study provides a granular analysis of the most frequently prescribed antibiotics, enabling the identification of antibiotics accounting for 90% of the total consumption and thus warranting focused stewardship efforts (Bergman et al.,1998). These insights further allow for the development of targeted interventions to optimize prescribing practices for these high-volume agents, thereby maximizing the mitigation effect of stewardship initiatives on overall antibiotic consumption and antimicrobial resistance. Furthermore, identifying these key antibiotics can be useful for healthcare systems to prioritize resources for antimicrobial susceptibility testing and surveillance, which can aid in refining empiric treatment guidelines and promote the use of narrower-spectrum agents when clinically appropriate (Ajulo and Awosile, 2024).

The in-hospital mortality rate of 26% (247/951) observed in this study reflects the high clinical severity of COVID-19 in a rural Indian tertiary care setting. This figure is consistent with reports from other high-burden regions in India, such as the 23.84% mortality rate documented during the pandemic’s second wave in Delhi (Katoch et al., 2022). The substantial fatality rate in this cohort is largely driven by critical respiratory and systemic complications, as 56% of patients developed ARDS and 21% progressed to septic shock. These clinical outcomes align with evidence from other Indian cohorts indicating that disease progression and the severity of systemic inflammation are primary determinants of mortality (Asirvatham et al., 2020; Rana et al., 2023). Furthermore, demographic factors were significant, with 84% of deaths occurring in patients over 40 years of age, echoing findings that advanced age and the presence of underlying comorbidities significantly escalate the risk of death in Indian hospital settings (Rana et al., 2023). The challenges of managing these severe cases are often amplified in rural areas, where late-stage patient presentation and disparities in healthcare access contribute to higher fatality rates compared to better-resourced urban centers (Asirvatham et al., 2020). While our findings highlight serious concerns regarding empirical antibiotic use in resource-constrained settings, they should be interpreted in the context of the single-center design and the unique challenges of the COVID-19 pandemic.

### Strengths and limitations

The main strength of our study lies in its comprehensive analysis of antibiotic prescribing patterns during the COVID-19 pandemic within an Indian tertiary care center, offering valuable insights into the factors influencing antibiotic prescription in a high-burden setting. Specifically, the detailed examination of clinical, biochemical, and demographic factors associated with antibiotic prescriptions contributes significantly to understanding the complexities of antimicrobial stewardship in such environments. The long duration and large sample size further enhance the representativeness of the findings.

The study’s limitations include its single-center, retrospective design, which may limit generalizability and introduce data biases. A major limitation of this study is the complete absence of microbiological culture data. Due to resource constraints during the COVID-19 pandemic, cultures were not sent for any patient. Consequently, we could not confirm bacterial co-infection or evaluate the appropriateness of antibiotic prescriptions. This highlights the urgent need for improved diagnostic capacity in resource-limited settings. The study was conducted during the extraordinary circumstances of the COVID-19 pandemic, when clinical practices, resource availability, and treatment protocols were constantly evolving.

## Conclusions

This study in a resource-constrained Central Indian setting found very high empirical broad-spectrum antibiotic use, with 90% of hospitalized COVID-19 patients receiving antibiotics. Prescribing relied heavily on WHO “Watch” agents (amoxicillin-clavulanate, doxycycline, azithromycin), which comprised the predominant DU90% segment. In the absence of cultures, elevated CRP was the strongest predictor of antibiotic use, highlighting diagnostic uncertainty as a key driver. These findings support strengthening diagnostic capacity and context-specific antimicrobial stewardship aligned with WHO AWaRe guidance to reduce unnecessary antibiotic exposure in similar resource-limited settings.

## Data Availability

The raw data supporting the conclusions of this article will be made available by the authors, without undue reservation.
