# Supplementary material for: Empirical antibiotic prescribing in COVID-19 patients: patterns and predictors in a resource-constrained Central Indian setting
Source: Front Antibiot. 2026 May 20;5:1826199. doi: 10.3389/frabi.2026.1826199 (PMC13231588; doi:10.3389/frabi.2026.1826199)
Supplement: Supplementary file 1 [file Table1.docx]

Supplement Table 1. WHO Ordinal Scale for Clinical Status of Patients (n = 951)

| **S.no.** | **Score** | **Patient status** | **Description** | **n (%)** |
| --- | --- | --- | --- | --- |
| 1 | 1 | **Ambulatory** | No limitation of activities | 72 (8) |
| 2 | 2-5 | **Mild distress** |  |  |
|  | 2 |  | Limitation of activities | 76 (8) |
|  | 3 |  | Hospitalized, No Oxygen Therapy | 82 (9) |
|  | 4 |  | Oxygen by mask or nasal Prongs | 101 (11) |
|  | 5 |  | Non-Invasive ventilation or High flow Oxygen | 68 (7) |
| 3 | 6-8 | **Severe** |  |  |
|  | 6 |  | Intubation and Mechanical Ventilation | 150 (16) |
|  | 7 |  | Ventilation + additional organ support –Vasopressors, ECMO, Renal replacement therapy | 153 (16) |
|  | 8 |  | Death | 249 (25) |
